# Spondylodiscitis in Children: A Retrospective Study and Comparison With Non-vertebral Osteomyelitis

**DOI:** 10.3389/fped.2021.727031

**Published:** 2021-10-21

**Authors:** Marco Roversi, Gianluca Mirra, Antonio Musolino, Domenico Barbuti, Laura Lancella, Daniele Deriu, Carlo Iorio, Alberto Villani, Marco Crostelli, Osvaldo Mazza, Andrzej Krzysztofiak

**Affiliations:** ^1^Academic Department of Pediatrics, Bambino Gesù Children's Hospital, IRCSS, Rome, Italy; ^2^Department of Imaging, Bambino Gesù Children's Hospital, IRCSS, Rome, Italy; ^3^Paediatric and Infectious Disease Unit, Academic Department of Pediatrics, Bambino Gesù Children's Hospital, IRCSS, Rome, Italy; ^4^Spine Surgery Unit, Department of Surgery and Transplantations, Bambino Gesù Children's Hospital, IRCSS, Rome, Italy; ^5^Department of Emergency, Acceptance and General Pediatrics, Bambino Gesù Children's Hospital, IRCSS, Rome, Italy

**Keywords:** spondylodiscitis, osteomyelitis, pediatrics, children, vertebra, tuberculosis

## Abstract

**Objectives:** The aim of this study is to provide new data on pediatrics spondylodiscitis for an optimal clinical management of this site-specific osteomyelitis.

**Methods:** We reported 48 cases of pediatric spondylodiscitis and made three comparisons between: (1) tubercular and non-tubercular cases; (2) patients aged more or less than 5 years; (3) children with spondylodiscitis and 62 controls with non-vertebral osteomyelitis.

**Results:** A higher rate of sequelae was reported in patients with tubercular spondylodiscitis, but no significant differences were noted at the cut-off of 5 years of age. Compared to non-vertebral osteomyelitis, pediatric spondylodiscitis affects younger children of both genders, usually presenting with afebrile back pain, and requiring longer time to admission, hospitalization, and antibiotic therapy.

**Conclusion:** Pediatric spondylodiscitis is an insidious disease with a non-specific presentation in childhood and peculiarities of its own. However, when clinical remission is obtained by an early start of broad-spectrum antibiotics, prolonging the therapy does not improve, nor worsens, the outcome. Surgical management is mandatory in case of vertebral instability and neurological signs but can be avoided when the infection is promptly treated with antibiotic therapy.

## Introduction

Pediatric spondylodiscitis (PSD) is a rare pathological entity that encompasses both infectious discitis and vertebral osteomyelitis, with or without an associated soft-tissue abscess. In the past, these two conditions were distinguished according to bone involvement, with the former found to be more common in children younger than 5 years owing to the relative abundance of blood vessels both in the cartilaginous vertebral endplate and within the growing vertebral body, where microorganisms or emboli are cleared by the several arterial anastomoses (1, 2). With the advent of Magnetic Resonance Imaging (MRI), early inflammatory edema of the disk-adjacent bones became evident and the distinction between infectious discitis and vertebral osteomyelitis became more subtle, with the two being now considered different stages of the same pathological process (3). However, recent data showed an age-dependent distribution of both the symptoms and etiology of this disease that may revalorize the old anatomical classification from a clinical point of view (4).

According to the most recent estimate, ~3% of all osteoarticular infections admitted to the pediatric orthopedic surgery unit were PSD (5). In our case series, PSD accounts for 15% of all cases of pediatric osteomyelitis in a period of 20 years [data not published]. Considering an incidence of pediatric osteomyelitis of 7–13 cases per 100,000 per year (6, 7), the estimate for PSD would be of 2–4 cases per 1,000,000 per year. Owing to its rarity and non-specific presentation with pain, whose nature and location is inadequately described by younger children, the diagnosis of PSD is often missed, with consequent delay of treatment and development of sequelae. Furthermore, in most cases current diagnostic tools do not provide a specific etiology, thus impairing targeted antibiotic therapies. Unlike in adults (8), no guidelines supported by clinical evidence are available on the management of PSD, including the duration and type of antibiotic therapy to administer. This is often reflected by the later development of severe complications, such as spinal deformities and epidural abscesses, which affect 4–38% of PSD cases and require urgent spine surgery (9).

In this retrospective study we report the demographic characteristics and clinical course of 48 cases of pediatric spondylodiscitis, and we compared them with controls with osteomyelitis to uncover significant risk factors and provide data for the development of pediatric guidelines on the management of this site-specific osteomyelitis.

## Methods

We retrospectively reviewed 48 cases of pediatric spondylodiscitis (PSD), admitted at the Bambino Gesù Children's Hospital (Rome, Italy), between January 2008 and November 2020. We included all patients with a clinical and radiological diagnosis of spondylodiscitis, with or without a microbiological positivity. The following demographic, clinical, hematic, and radiological variables were reported: age at onset; age groups (lower than 1 year; between 1 and 5 years, over 5 years); gender; clinical presentation (either with back pain or coxalgia or limp or other); presence or absence of fever and pain or tenderness at admission; history of trauma; coexisting comorbidities; blood parameters, including white blood cells (WBC, cells/mm^3^), neutrophils (% of WBC), erythrocyte sedimentation rate (ESR, mm/h), C-Reactive Protein (CRP, mg/dl); microbiologic sample obtained (either blood culture or bone biopsy or PCR assay or other); etiology (when available); medical imaging, including X-ray, ultrasound, computerized tomography scan (CT-scan), magnetic resonance imaging (MRI), scintigraphy; presence of cellulitis or myositis and soft-tissue abscess; site of infection (vertebral region); length of intravenous (IV), oral (OS) and total (both IV and OS) antibiotic therapy; days of hospitalization; months of follow-up; development of sequelae.

Within the same cohort, we compared patients with or without tuberculous spondylodiscitis, diagnosed *via* standard culture or immunoreactivity test (Mantoux or Interferon Gamma Release Assay, IGRA) and patients with more or less than 5 years of age (1, 2). Moreover, we compared these PSD cases with a cohort of matched controls with non-vertebral (non-PSD) osteomyelitis. The controls were chosen among the patients aged <18 years admitted at the Bambino Gesù Children's Hospital during the same period of the cases with a diagnosis of pediatric osteomyelitis of the proximal femur, hip bone (ileus, ischium, and/or pubis), shoulder (scapula, clavicle, and/or acromion), and proximal homer. Finally, we performed a linear and binary logistic regression adopting the length of hospitalization and sequelae, respectively, as the dependent variables of the analysis.

The software IBM SPSS version 23.0 was used for statistical analysis. Continuous normally distributed variables were expressed as means and standard deviations and analyzed with the Student *t*-test. Continuous non-normally distributed variables were expressed as medians and ranges and analyzed with the Mann-Whitney *U*-test. Categorical variables were expressed as proportions and percentages and analyzed with the Chi-squared test or Fisher exact test (when appropriate). A *p* < 0.05 was considered statistically significant.

## Results

The demographic and clinical characteristics of patients with PSD and tPSD are outlined in Table 1. The same characteristics of patients aged more or less than 5 years are outlined in Table 2.

**Table 1:** Demographic and clinical characteristics of patients with and without tubercular PSD (tPSD).

	**All included**	**Excluding tPSD**	**Only tPSD**	***p-value*** **^a^**
Total sample—no.	**48^b^**	**42^b^**	**6^b^**	
Age (years)—median (range)	**3.8** (0.3–17.7)	**2.8** (0.3–16.9)	**13.5** (6.8–17.7)	**0.007**
Age distribution—no. (%)				
<1 year	**4** (8%)	**4** (10%)	**0** (0%)	-
1 to 5 years	**22** (46%)	**22** (52%)	**0** (0%)	-
>5 years	**22** (46%)	**16** (38%)	**6** (100%)	**0.006**
Sex—no. (%)				
Male	**23** (48%)	**20** (48%)	**3** (50%)	0.625
Female	**25** (52%)	**22** (52%)	**3** (50%)	-
Male to female ratio	**1:1.1**	**1:1.1**	**1:1**	-
Clinical presentation				
Back pain	**20** (42%)	**20** (48%)	**0** (0%)	-
Coxalgia	**5** (10%)	**5** (12%)	**0** (0%)	-
Limp	**5** (10%)	**5** (12%)	**0** (0%)	-
Other	**18** (38%)	**12** (28%)	**6** (100%)	0.002
Fever	**22** (46%)	**19** (45%)	**3** (50%)	0.582
Pain/tenderness	**46** (96%)	**42** (100%)	**4** (67%)	**0.013**
History of trauma	**8** (17%)	**7** (17%)	**1** (17%)	0.742
Comorbidities at presentation	**19** (40%)	**16** (38%)	**3** (50%)	0.658
Time to admission (days)—median (range)	**21.5** (2–148)	**20.5** (2–148)	**60** (3–133)	0.170
Blood parameters				
White blood cells—mean (SD)	**11167** (4203)	**11440** (4246)	**9167** (3577)	0.195
Neutrophils %—mean (SD)	**0.55** (0.57)	**0.52** (0.18)	**0.73** (0.09)	** <0.001**
ESR (mm/h)—mean (SD)	**43.8** (25.7)	**40.9** (24.3)	**69** (28)	0.136
CRP (mg/dl)—median (range)	**2** (0–30.7)	**1.8** (0–30.7)	**2.9** (1.4–12)	0.226
IgA (g/l)—median (range)	**1.03** (0.1–7.34)	**0.94** (0.1–7.34)	**4.30** (3.54–6.18)	**0.002**
IgG (g/l)—median (range)	**10.6** (2.3–34.6)	**9.7** (2.3–20.6)	**20.6** (17.7–34.6)	**0.002**
IgM (g/l)—mean (SD)	**1.1** (0.48)	**1.1** (0.27)	**1.01** (0.51)	0.585
Microbiological positivity (overall)	**13** (27%)	**8** (19%)	**5** (83%)	**0.004**
Radiological imaging				
X-ray	**43** (90%)	**38** (90%)	**5** (83%)	0.503
Ultrasound of hip joint	**4** (8%)	**4** (10%)	**0** (0%)	-
CT-scan	**18** (38%)	**13** (41%)	**5** (83%)	**0.022**
MRI	**46** (96%)	**41** (98%)	**5** (83%)	0.237
Scintigraphy	**23** (48%)	**20** (48%)	**3** (50%)	0.625
Biopsy	**10** (21%)	**6** (14%)	**4** (67%)	**0.013**
Perilesional cellulitis/myositis	**7** (15%)	**7** (17%)	**0** (0%)	-
Soft-tissue abscess	**16** (33%)	**11** (26%)	**5** (83%)	**0.012**
Site of infection (vertebral region)				
Cervical	**3** (6%)	**3** (7%)	**0** (0%)	-
Thoracic	**5** (11%)	**3** (7%)	**2** (33%)	0.111
Thoraco-lumbar	**4** (8%)	**2 (**5%)	**2** (33%)	0.071
Lumbar	**21** (44%)	**21** (50%)	**0** (0%)	-
Lumbo-sacral	**12** (25%)	**10** (24%)	**2** (33%)	0.631
Sacral	**3** (6%)	**3** (7%)	**0** (0%)	-
IV therapy (days)—median (range)	**27.5** (0–87)	**27** (0−64)	**53** (0−87)	0.190
<14 days	**4** (8%)	**3** (7%)	**1** (17%)	0.425
>14 days	**44** (92%)	**39** (93%)	**5** (83%)	-
OS therapy (days)—median (range)	**23** (0–526)	**22** (0−51)	**419** (62–526)	** <0.001**
<14 days	**9** (19%)	**9** (21%)	**0** (0%)	0.320
>14 days	**39** (81%)	**33** (79%)	**6** (100%)	-
Total antibiotic therapy (days)—median (range)	**51** (21–561)	**49** (21–86)	**482** (112–562)	** <0.001**
<6 weeks	**41** (86%)	**41** (98%)	**0** (0%)	** <0.001**
>6 weeks	**7** (14%)	**1** (2%)	**6** (100%)	-
Hospitalization (days)—median (range)	**30** (13–265)	**30** (13–265)	**53.5** (21–91)	**0.037**
Follow-up (months)—median (range)	**12** (0–60)	**11** (0–60)	**18** (3–36)	0.255
Sequelae^c^	**9** (19%)	**4** (10%)	**5** (83%)	**<0.001**

a *Comparison between patients with or without tPSD*.

b *Data were calculated accounting for missing values*.

c *Mainly kyphosis (n = 5) and scoliosis (n = 2), followed by rigidity (n = 1) and gibbus (n = 1)*.

*Bold values are highlight the most significant ones in terms of absolute value*.

**Table 2:** Demographic and clinical characteristics of patients aged more or less than 5 years.

	**Aged <5 years**	**Aged >5 years**	***p-value*** **^a^**
Total sample—no.	**26^b^**	**22^b^**	
Tubercular PSD—no.	**0** (0%)	**6** (27%)	**0.006**
Age (years)—median (range)	**1.6** (0.3–4.2)	**13.1** (6.3–17.7)	**<0.001**
Sex—no. (%)			
Male	**11** (42%)	**12** (55%)	0.563
Female	**15** (58%)	**10** (45%)	-
Male to female ratio	**1:1.3**	**1.2:1**	-
Clinical presentation			
Back pain	**10** (38%)	**10** (45%)	0.770
Coxalgia	**2** (8%)	**3** (14%)	0.649
Limp	**5** (19%)	**0** (0%)	-
Other	**9** (35%)	**9** (41%)	0.768
Fever	**11** (42%)	**11** (50%)	0.772
Pain/tenderness	**26** (100%)	**20** (91%)	0.205
History of trauma	**4** (15%)	**4** (18%)	0.548
Comorbidities at presentation	**9** (35%)	**9** (41%)	0.768
Time to admission (days)—median (range)	**20** (5–111)	**28** (2–148)	0.175
Blood parameters			
White blood cells—mean (SD)	**12066** (3922)	**10144** (4366)	0.119
Neutrophils %—mean (SD)	**0.44** (0.14)	**0.67** (0.14)	**<0.001**
ESR (mm/h)—mean (SD)	**43** (26)	**44** (27)	0.899
CRP (mg/dl)—median (range)	**1.5** (0–30.7)	**3.2** (0.1–16.3)	**0.028**
IgA (g/l)—median (range)	**0.76** (0.1–2.01)	**1.75** (0.79–7.34)	**<0.001**
IgG (g/l)—median (range)	**9.7** (2.3–14.7)	**10.9** (8.5–34.6)	**0.025**
IgM (g/l)—mean (SD)	**1.07** (0.46)	**1.14** (0.52)	0.665
Microbiological positivity (overall)	**6** (23%)	**10** (45%)	**0.131**
Blood culture	**2** (33%)	**4** (40%)	0.392
Bone biopsy	**0** (0%)	**4** (40%)	-
PCR assay	**0** (0%)	**1** (10%)	-
Others	**4** (67%)	**1** (10%)	0.357
Aetiology			
*S.aureus*	**1** (17%)	**4** (40%)	0.165
*M.tuberculosis*	**0** (0%)	**5** (50%)	**0.006**
*K.kingae*	**4** (66%)	**0** (0%)	0.114
*S.intermedius*	**0** (0%)	**1** (10%)	-
*M.catarrhalis & H.influenzae^c^*	**1** (17%)	**0** (0%)	-
Radiological imaging			
X-ray	**25** (96%)	**18** (81%)	0.165
Ultrasound of hip joint	**3** (12%)	**1** (45%)	0.614
CT-scan	**7** (27%)	**11** (50%)	0.138
MRI	**25** (96%)	**21** (95%)	0.712
Scintigraphy	**12** (46%)	**11** (50%)	0.509
Biopsy	**2** (8%)	**8** (36%)	**0.029**
Perilesional cellulitis/myositis	**5** (19%)	**2** (9%)	0.429
Soft-tissue abscess	**7** (27%)	**9** (41%)	0.366
Site of infection (vertebral region)			
Cervical	**1** (4%)	**2** (9%)	0.587
Thoracic	**2** (8%)	**3** (14%)	0.649
Thoraco-lumbar	**2** (8%)	**2** (9%)	0.629
Lumbar	**12** (46%)	**9** (41%)	0.776
Lumbo-sacral	**8** (31%)	**4** (18%)	0.505
Sacral	**1** (4%)	**2** (9%)	0.587
IV therapy (days)—median (range)	**28** (9–64)	**28** (0–87)	0.885
<14 days	**1** (4%)	**3** (14%)	0.320
>14 days	**25** (96%)	**19** (86%)	-
OS therapy (days)—median (range)	**23** (0–43)	**28** (5–526)	0.192
<14 days	**6** (23%)	**4** (21%)	0.735
>14 days	**20** (77%)	**18** (69%)	-
Total antibiotic therapy (days)—median (range)	**49** (28–86)	**54** (21–561)	0.233
<6 weeks	**25** (96%)	**16** (73%)	**0.038**
>6 weeks	**1** (4%)	**6** (27%)	**-**
Hospitalization (days)—median (range)	**31** (14–265)	**30** (13–91)	0.836
Follow-up (months)—median (range)	**13** (0–60)	**12** (1–40)	0.488
Sequelae^d^	**4** (10%)	**5** (83%)	0.713

a *Comparison between patients with or without tPSD*.

b *Data were calculated accounting for missing values*.

c *Isolated from hypopharyngeal aspirate*.

d *Mainly kyphosis (n = 5) and scoliosis (n = 2), followed by rigidity (n = 1) and gibbus (n = 1)*.

*Bold values are highlight the most significant ones in terms of absolute value*.

### Demographics

Our sample consisted of 48 patients, six of which had a tuberculous PSD. The median age was 3.8 years (range 0.3–17.7). Twenty-six patients were <5 years and 22 patients were >5 years old. Patients with tPSD were significantly older than those with PSD, with a median age of 13.5 years (range 6.8–17.7). All patients with tPSD were older than 5 years. The ratio of males and females between the groups of patients with PSD or tPSD and aged less or more than 5 years did not differ significantly, with an approximately equal representation of both genders in all subgroups.

### Clinical Presentation

Most patients presented with back pain (42%), followed by coxalgia (10%) and limp (10%). None of the patients with tPSD had such clinical presentation. About half the patients presented fever at admission (46%) and almost all lamented pain and/or tenderness (96%). Only four patients with tPSD (67%) presented with pain and/or tenderness. Seventeen percentage of all patients had a history of trauma and up to 40% had some comorbidity at presentation, namely asplenia, IgA deficit, recent appendicectomy, homozygous MTHFR mutation, left cephalic vein thrombophlebitis, hypertrophic cardiomyopathy, and Poland syndrome. The median time to admission was 21.5 days. We did not find significant differences in the clinical presentation between patients aged more or less than 5 years.

### Laboratory Workup

The laboratory workup showed a mean WBC count of 11,167 cells/mm^3^ (SD ± 4,203), which did not differ significantly between PSD and tPSD, and a mean neutrophil percentage of 55%, with a significantly higher value in tPSD patients (73 vs. 52% of PSD patients, *p* < 0.001) and in children older than 5 years (67 vs. 44% of children younger than 5 years, *p* < 0.001). The erythrocyte sedimentation rate (ESR) was increased in all patients, with a mean value of 43.8 mm/h (SD ± 25.7). C-reactive protein (CRP) levels were only slightly elevated (median value 2 mg/dl), although very high in a few subjects (range 0–30.7). A significantly higher value of CRP was demonstrated in patients >5 years (median value 3.2 vs. 1.5 mg/dl of patients <5 years, *p* = 0.028). The levels of IgG and IgA, but not IgM, appeared significantly elevated in patients with tPSD and, not surprisingly, in those >5 years (see Tables 1, 2 for data). Samples of blood or other biological material (saliva, urine or pus from the infected site) were sent for bacterial culture in all patients but tested positive in only 16 cases (33%). The rate of positivity was significantly higher in patients with tPSD (83 vs. 19% of PSD patients, *p* = 0.004). *S.aureus* (*n* = 5, four of which were MRSA), and *M.tuberculosis* (*n* = 5) were the most commonly isolated pathogens in all PSDs, followed by *K.kingae* (*n* = 4), *S.intermedius* (*n* = 1), *M.catarrhalis* and *H.influenzae* (*n* = 1).

### Radiological Imaging

Most patients initially underwent standard X-ray imaging, oftentimes during the Emergency Department evaluation, with anterior-posterior and lateral projections (90%), followed by magnetic resonance imaging (MRI) with contrast medium (96%). Scintigraphy and CT-scan were performed in 48 and 38% of cases, respectively. The CT-scan was more often required in case of tPSD (83 vs. 41% of PSD patients, *p* = 0.022). When, in the lack of a microbiological isolate, intravenous broad-spectrum antibiotic therapy did not improve laboratory tests after 2 weeks, a CT-guided biopsy of the lesion was performed (21%), more commonly in tPSD (67 vs. 14% of PSD patients, *p* = 0.013) and in patients >5 years (50 vs. 27% of patients <5 years, *p* = 0.029). The most common site of the infection were the lumbar (44%) and lumbo-sacral (25%) tracts of the vertebral column. Sixteen patients had involvement of adjacent tracts. Perilesional cellulitis or myositis was observed in 15% of patients. Soft-tissue abscesses were observed in 33% of cases, though they were more common in tPSD patients (83 vs. 26% of PSD patients, *p* = 0.012).

### Treatment, Follow-Up, and Sequelae

**Figure 1:**
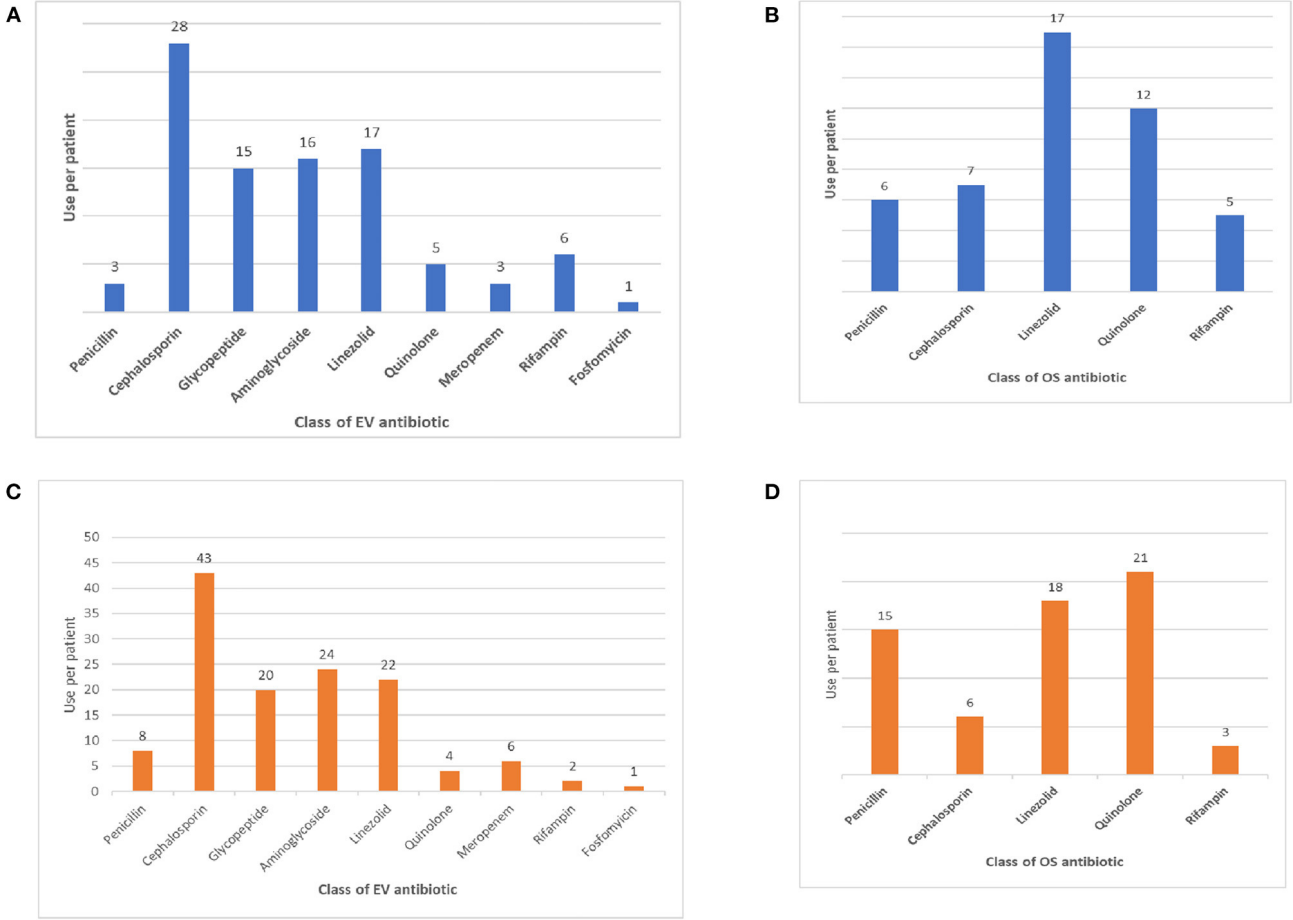
(**A)** Intravenous antibiotic therapy in PSDs. **(B)** Oral antibiotic therapy in PSDs. **(C)** Intravenous antibiotic therapy in non-PSD osteomyelitis. **(D)** Oral antibiotic therapy in non-PSD osteomyelitis.

The classes of antibiotics administered are shown in Figure 1.

All patients were started with intravenous broad-spectrum antibiotic therapy on admission, later modified according to the patient's clinical response and isolates. The most administered IV antibiotics were cephalosporins (*n* = 28), followed by linezolid (*n* = 17), aminoglycosides (*n* = 16) and glycopeptides (*n* = 15). The most common oral antibiotics administered were linezolid (*n* = 17) and quinolones (*n* = 12). The median time of IV and OS antibiotic therapy was 27.5 days (range 0–87) and 23 days (range 0–526). The median time of total antibiotic therapy was 51 days (21–561). As expected, the median time of OS antibiotic therapy differed significantly between patients with PSD and tPSD (419 vs. 22 days of PSD patients, *p* < 0.001), thus driving the corresponding increase of total antibiotic therapy in the latter subgroup. The length of IV or OS antibiotic therapy did not differ between subjects older and younger than 5 years, although a significantly higher fraction of patients <5 years were treated for < 6 weeks (96 vs. 73% of patients <5 years, *p* = 0.038). All patients were treated with immobilization in cast braces for an average period of 4 months (range 3–6 months), followed by another 4 months on average (range 3–8 months) of semi-rigid bracing (data not shown).

The median length of hospitalization was 30 days (range 13–265) and was higher for patients with tPSD (53.5 vs. 30 days of PSD patients, *p* = 0.037). The median time of follow-up was 12 months (range 0–60). All patients not lost to follow-up were studied with contrast-enhanced MRI at 4, 8, 12 and 24 weeks and CT-scan at 6, 12, and 18 weeks from the start of treatment, to check for damage of the spinal cord and/or merging of affected adjacent vertebrae. Sequelae, mainly kyphosis at a Cobb angle of more than 25° (*n* = 5) and scoliosis (*n* = 2), followed by rigidity (*n* = 1) and gibbus (*n* = 1), were reported in 19% of all cases, with a higher incidence in tPSD patients (83 vs. 10% of PSD patients, *p* < 0.001). No neurological complaint was later reported. All patients survived through the follow-up.

### Comparison With Non-PSD Osteomyelitis Controls

The comparison between patients with PSD and controls with non-PSD osteomyelitis is shown in Table 3. The total number of matched non-PSD osteomyelitis were 62. The median age of this group was 4.5 years (range 0.02–17.9), with a higher male to female ratio than PSD patients (73 vs. 48% of males, *p* = 0.008). Most patients were older than 5 years (85 vs. 46% of PSD patients, *p* < 0.001). Fever was significantly more frequent in patients with non-PSD osteomyelitis (77 vs. 46% of PSD patients, *p* = 0.001). This may have led to an earlier referral to the Emergency Department, as the time to admission was also significantly lower in this subgroup (5 vs. 21.5 days of PSD patients, *p* < 0.001). Patients with non-PSD osteomyelitis also showed a higher count of WBC (13,516 vs. 11,167 cells/mm^3^ of PSD patients, *p* = 0.022) and increased CRP levels (7.5 vs. 2 mg/dl of PSD patients, *p* < 0.001). The microbiological positivity of blood cultures was also higher in this subgroup compared to the former (80 vs. 31%, *p* = 0.002) with *S.aureus* being the most commonly isolated pathogen (76 vs. 31%, *p* = 0.001). Conversely, in PSD patients *M.tuberculosis* (31 vs. 3%, *p* = 0.165) and *K.kingae* (26 vs. 0%, *p* = 0.034) were more isolated than in non-PSD osteomyelitis. The hip joint ultrasound (72 vs. 8%, *p* < 0.001) and CT-scan (11 vs. 38%, *p* = 0.001) were more frequently performed in patients with non-PSD osteomyelitis. Perilesional cellulitis was also more frequently detected in this subgroup (50 vs. 15%, *p* < 0.001). The duration of IV antibiotic therapy was shorter in patients with non-PSD osteomyelitis (20.5 vs. 27.5 days, *p* = 0.009), often lasting less than 14 days (23 vs. 8%, *p* = 0.045). Consequently, the length of hospitalization was also longer than in PSD patients (22 vs. 30 days, *p* = 0.001).

**Table 3:** Demographic and clinical characteristics of patients with PSDs and controls with osteomyelitis.

	**PSDs**	**Controls**	***p-value*** **^a^**
Total sample—no.	**48^b^**	**62^b^**	
Age (years)—median (range)	**3.8** (0.3–17.7)	**4.5** (0.02–17.9)	**0.691**
Age distribution—no. (%)			
<1 year	**4** (8%)	**3** (5%)	0.456
1 to 5 years	**22** (46%)	**6** (10%)	**<0.001**
>5 years	**22** (46%)	**53** (85%)	**<0.001**
Sex—no. (%)			
Male	**23** (48%)	**45** (73%)	**0.008**
Female	**25** (52%)	**17** (27%)	-
Male to female ratio	**1:1.1**	**1:0.4**	-
Clinical presentation			
Back pain	**20** (42%)	**4** (6%)	**<0.001**
Coxalgia	**5** (10%)	**19** (31%)	**0.011**
Limp	**5** (10%)	**6** (10%)	1.000
Other	**18** (38%)	**33** (53%)	0.101
Fever	**22** (46%)	**48** (77%)	**0.001**
Pain/tenderness	**46** (96%)	**61** (98%)	0.415
History of trauma	**8** (17%)	**9** (15%)	0.757
Comorbidities at presentation	**19** (40%)	**14** (23%)	0.088
Time to admission (days)—median (range)	**21.5** (2–148)	**5** (0–67)	**<0.001**
Blood parameters			
White blood cells—mean (SD)	**11167** (4203)	**13516** (5848)	**0.022**
Neutrophils %—mean (SD)	**0.55** (0.57)	**0.59** (0.2)	0.276
ESR (mm/h)—mean (SD)	**43.8** (25.7)	**46.7** (26.2)	0.720
CRP (mg/dl)—median (range)	**2** (0–30.7)	**7.5** (0–87)	**<0.001**
IgA (g/l)—median (range)	**1.03** (0.1–7.34)	**1.24** (0.05–3.56)	0.828
IgG (g/l)—median (range)	**10.6** (2.3–34.6)	**9.42** (1.7–28.26)	0.135
IgM (g/l)—mean (SD)	**1.1** (0.48)	**1.2** (0.49)	0.238
Microbiological positivity (overall)	**16** (33%)	**30** (48%)	0.124
Blood culture	**5** (31%)	**24** (80%)	**0.002**
Bone biopsy	**4** (25%)	**1** (3%)	0.165
PCR assay	**1** (6%)	**3** (10%)	0.631
Others	**6** (38%)	**2** (7%)	0.236
Aetiology			
*S.aureus*	**5** (31%)	**23** (76%)^**^	**0.001**
*M.tuberculosis*	**5** (31%)	**1** (3%)	0.165
*K.kingae*	**4** (26%)	**0** (0%)	**0.034**
*S.intermedius*	**1** (6%)	**0** (0%)	-
*M.catarrhalis & H.influenzae^c^*	**1** (6%)	**0** (0%)	-
*E.coli & E.faecalis*	**0** (0%)	**1** (3%)	-
Streptococci spp.^*^	**0** (0%)	**2** (7%)	-
*S.hominis*	**0** (0%)	**2** (7%)	-
*C.albicans*	**0** (0%)	**1** (3%)	-
Radiological imaging			
X-ray	**43** (90%)	**60** (96%)	0.236
Ultrasound of hip joint	**4** (8%)	**45** (72%)	**<0.001**
CT-scan	**18** (38%)	**7** (11%)	**0.001**
MRI	**46** (96%)	**61** (98%)	0.579
Scintigraphy	**23** (48%)	**27** (44%)	0.648
Biopsy	**10** (21%)	**6** (9%)	0.100
Perilesional cellulitis/myositis	**7** (15%)	**31** (50%)	**<0.001**
Soft-tissue abscess	**16** (33%)	**32** (52%)	0.055
multicolumn4@l Site of infection			
Vertebrae	**48** (6%)	**-**	-
Femur	**-**	**26** (42%)	-
Hip bone	**-**	**23** (37%)	-
Shoulder	-	**7** (11%)	-
Homer	-	**6** (10%)	-
IV therapy (days)—median (range)	**27.5** (0–87)	**20.5** (0–112)	**0.009**
<14 days	**4** (8%)	**14** (23%)	**0.045**
>14 days	**44** (92%)	**48** (77%)	-
OS therapy (days)—median (range)	**23** (0–526)	**18.5** (0–360)	0.174
<14 days	**9** (19%)	**17** (27%)	0.289
>14 days	**39** (81%)	**45** (73%)	-
Total antibiotic therapy (days)—median (range)	**51** (21–561)	**42** (1–360)	**0.007**
<6 weeks	**41** (86%)	**28** (45%)	**<0.001**
>6 weeks	**7** (14%)	**34** (55%)	-
Hospitalization (days)—median (range)	**30** (13–265)	**22** (6–246)	**0.001**
Follow-up (months)—median (range)	**12** (0–60)	**7.5** (0–60)	0.209
Sequelae^d^	**9** (19%)	**15** (24%)	0.465

a *Comparison between patients with PSD and controls with osteomyelitis*.

b *Data were calculated accounting for missing values*.

c *Isolated from hypopharyngeal aspirate*.

d *Mainly kyphosis (n = 5) and scoliosis (n = 2), followed by rigidity (n = 1) and gibbus (n = 1)*.

* *Of which, 1 S.pneumoniae and 1 S.pyogenes*.

** *3 of which were MRSA*.

*Bold values are highlight the most significant ones in terms of absolute value*.

### Multivariate Analysis

We performed linear and binary logistic regression adopting the length of hospitalization and sequelae, respectively, as the dependent variables (data not shown). This analysis did not yield significant results. Among the independent variables evaluated, we included age, gender, tuberculous PSD, signs and symptoms at presentation, fever, trauma, comorbidities, blood parameters, perilesional cellulitis and/or myositis, soft-tissue abscess, length of IV, OS and/or total antibiotic therapy, sequelae. None of these significantly predicted the length of hospitalization and sequelae when one was adjusted for the other.

## Discussion

Tuberculous PSD (tPSD) deserves a special mention among the PSDs, owing to its specificity of presentation, diagnosis, and treatment. All patients with tPSD received different combinations of oral isoniazid, rifampicin, ethambutol, or pyrazinamide. In our series, we recognized a peak of tPSD during adolescence. All signs and symptoms associated with PSD were lacking in tPSD patients at presentation. Back pain was never reported. Clinical suspicion was usually risen months afterwards, because of an unexplained fever or a sudden-onset kyphosis. Consequently, a higher rate of sequelae was recorded in tPSD patients, who often required surgical intervention (data not shown).

The comparison between patients at the cut-off of 5 years of age did not show significant differences in the presentation, diagnosis, treatment, and outcomes of PSD, differently from what is suggested in the literature (1, 2). However, a slight elevation of neutrophils and CRP levels was noted in patients older than 5 years. Moreover, younger patients required a longer total antibiotic treatment, possibly owing to a more cautious approach to this more fragile population.

The diagnostic and therapeutic course of non-PSD osteomyelitis proved to be burdened by a minor delay compared to PSD. Namely time to admission, hospitalization, and antibiotic therapy duration were significantly lower in patients with non-PSD osteomyelitis. This may be due to differences in the origin of the infection that could not be overlooked by the case-control design, but also to an intrinsic higher severity of PSD. Compared to PSD, non-PSD osteomyelitis involved older children, with no clear gender prevalence, who might have better report their symptoms and tolerate the diagnostic investigations, such as the ultrasound imaging, thus accelerating the diagnosis. However, the more frequent non-specific back pain onset and reduced rate of fever and leukocytosis observed in PSDs may also be responsible of the diagnostic delay. Furthermore, the microbiological positivity of cultures, though low as reported in the literature (10), was higher (not significantly) in non-PSD osteomyelitis (with *S.aureus* being the single most reported pathogen). This might also explain the increased use of CT-guided biopsy of the affected vertebra in PSD patients, as this exam was performed after 2 weeks of broad-spectrum antibiotic therapy in patients without a positive culture and an absent or slow clinical improvement. These findings are not paralleled in the adult population, where higher positivity rates are observed (11), suggesting that blood cultures should be obtained early, both in the pyretic and afebrile patient—with strong clinical suspicion—to avoid both therapeutic failure and invasive procedures.

In our study, the multivariate analysis did not identify any adjusted parameter to predict the duration of hospitalization and the onset of sequelae. More specifically, no predictor was significantly associated with the target outcome when adjusted for the tubercular etiology, thus remarking its role in the increase of both length of stay and the complications observed. Interestingly, the choice and duration of the antibiotic therapy, did not prove to negatively correlate with the onset of sequelae, as would be expected. This could mean that shorter courses of antibiotic treatment may be attempted in PSD, while monitoring the clinical and laboratory indices of improvement.

### Considerations on PSD Management

Despite the delayed management of PSD compared to adult spondylodiscitis (11), the outcome is still favorable in children who are treated aggressively with a broad-spectrum or targeted antibiotic therapy in a multidisciplinary environment, where spinal surgeons and pediatric infectivologists set together the therapeutic milestones to reach. At the moment of writing, no guidelines for the conservative and surgical treatments of PSD are yet available. Thus, the current pediatric practice is derived from the management of adult spondylodiscitis (12–14) and relies upon the experience of the treating clinicians. After clinical suspicion of PSD is risen, contrast-enhanced magnetic resonance imaging is the investigation of choice for the description of the infected site location and extension (3). As this is also the most sensitive technique to detect spinal involvement, we recommend its use both in the acute phase and in the follow-up of patients with PSD. To evaluate osteonecrosis or other bony sequelae and to guide the surgical intervention, CT scans are mandatory in PSD. Regarding the antibiotic therapy, most authors agree upon long periods—up to 6 weeks—of antibiotic therapy and bed rest (3, 15). This treatment should lead to complete sterilization of the infected site, as to allow a “clean” surgery when needed. During antibiotic therapy, immobilization in cast braces prevents back pain, vertebral collapse and consequent neurological complications, and secondary deformity of the spine, also allowing the patient to be mobilized. When acute neurologic impairment or severe kyphosis occurs, an open spinal decompression and stabilization should be performed as soon as possible. In the lack of urgency spinal surgery should be postponed until complete sterilization of the infected site is reached.

### Limitations

Our study has some limitations. First, the single-center design does not allow us to generalize our findings. Second, we reported data gathered from both the PSD and tPSD cohorts, although we also analyzed them separately. However, we believed that the “effect size” of the latter would be diluted by the former sample, given that only six patients had tPSD. Third, when we performed the case-control study, we adopted all osteomyelitis “proximal” to the vertebral column as surrogates of PSDs. However, there are significant anatomical differences between hips and shoulders, which may have disrupted the homogeneity of the control sample and introduced significant unwanted differences from the PSDs. Fourth, we only considered the clinically evident sequelae, while neglecting all radiographic “scars” of the vertebra that may persist for months or years in symptom-free patients.

## Conclusions

PSD is a rare and insidious disease of infancy, whose diagnosis and treatment is often delayed, due to a non-specific presentation in childhood. Broad-spectrum antibiotic treatment should be started soon after a microbiological sample is obtained and should be prolonged until clinical remission occurs. When this objective is reached early during hospitalization and confirmed by seriated MRI and/or CT scans, our study suggests that prolonging the antibiotic therapy does not improve, nor worsens, the outcome. Although our study provided interesting data on PSD, we believe that more multicenter studies are needed to consolidate, or refute, our management proposal for PSD.

## Data Availability Statement

The original contributions presented in the study are included in the article/supplementary material. Requests to access the datasets should be directed to marcrov@outlook.it.

## Ethics Statement

Ethical review and approval was not required for the study on human participants in accordance with the local legislation and institutional requirements. Written informed consent from the participants' legal guardian/next of kin was not required to participate in this study in accordance with the national legislation and the institutional requirements.

## Author Contributions

MR, GM, and AK conceptualized and designed the article. GM, AM, and DD data collection. MR data analysis. MR and GM drafted and wrote the manuscript. MR, GM, CI, OM, and AK performed literature review and interpretation of data. DB, LL, MC, and AV revised the manuscript for important intellectual content. All authors reviewed and accepted the final manuscript.

## Conflict of Interest

The authors declare that the research was conducted in the absence of any commercial or financial relationships that could be construed as a potential conflict of interest.

## Publisher's Note

All claims expressed in this article are solely those of the authors and do not necessarily represent those of their affiliated organizations, or those of the publisher, the editors and the reviewers. Any product that may be evaluated in this article, or claim that may be made by its manufacturer, is not guaranteed or endorsed by the publisher.
